# HCV Ag quantification as a one-step procedure in diagnosing chronic hepatitis C infection in Cameroon: the ANRS 12336 study

**DOI:** 10.7448/IAS.20.1.21446

**Published:** 2017-05-15

**Authors:** Léa Duchesne, Richard Njouom, Frédéric Lissock, Gishlaine Flore Tamko-Mella, Sandrine Rallier, Lila Poiteau, Alexandre Soulier, Stéphane Chevaliez, Guy Vernet, Nicolas Rouveau, Jean-Michel Pawlotsky, Pierre-Marie Girard, Karine Lacombe

**Affiliations:** ^a^ Institut Pierre Louis d’épidémiologie et de Santé Publique (IPLESP UMRS 1136), Sorbonne Universités, UPMC Univ Paris 06, Inserm, Paris, France; ^b^ Virology Department, Pasteur Center of Cameroun, Yaoundé, Cameroon; ^c^ National Reference Center for Viral Hepatitis B, C and delta, Department of Virology, Hôpital Henri Mondor, Inserm U955, Université Paris-Est, Créteil, France; ^d^ “Research in Resource-Limited Settings” Department, ANRS, Paris, France; ^e^ Service des maladies infectieuses et tropicales, Hôpital Saint-Antoine, AP-HP, Paris, France

**Keywords:** chronic hepatitis C, HCV core antigen, diagnosis, Sub-Saharan Africa, co-infection, HIV, hepatitis B virus

## Abstract

**Introduction**: The diagnostic procedure for chronic hepatitis C infection (CHC) usually combines anti-HCV antibody (HCV-*Ab*) and HCV-RNA measurement. Quantifying HCV core antigen (cAg) as a one-step procedure could shorten the diagnostic process. We aimed to assess the performance of cAg quantification in diagnosing CHC and how it is influenced by concomitant HIV or HBV infections.

**Methods**: The cAg was quantified by an automated assay (Abbott Diagnostics) in 465 HCV-*Ab* negative serum samples and 544 HCV-RNA positive serum samples (*n* = 1009) collected in patients from the Pasteur Center in Cameroon, some of whom were infected by HBV or HIV. Its performance was evaluated in comparison to the gold standard (ELISA or PCR) by estimating its sensitivity (Se) and specificity (Sp), and by comparing the area under ROC (AUROC) curves in each patient population: HCV mono-infected, HCV-HBV and HIV-HCV co-infected.

**Results**: Among the 465 HCV-*Ab* negative patients, 51 and 79 were HIV- and HBV-infected, respectively, whereas among the 544 patients with CHC, 27 and 28 were HIV- and HBV-infected, respectively. The Spearman *ρ* correlation coefficient between cAg and HCV-RNA was 0.75 (*p* < 0.00001). The assay had a sensitivity of 95.7% (95% CI: 93.2–97.5) and a specificity of 99.7% (95% CI: 98.1–10) in diagnosing CHC, corresponding to an AUROC of 0.99 (95% CI: 0.98–1.0). Being HIV- or HBV-infected did not impact the performance of cAg (Se = 96.4%, Sp = 96.2% and AUROC = 0.98 (95% CI: 0.95–1.0) in the HBV group, Se = 100%, Sp = 88.2% and AUROC = 0.99 (95% CI: 0.97–1.0) in the HIV group, *p* between AUROC = 0.69).

**Conclusions**: The cAg quantification displayed a high specificity and sensitivity for the diagnosis of CHC in Cameroon, and its performance was not significantly modified by a concomitant HIV or HBV infection. In the context of CHC elimination on a global scale, using cAg quantification as a screening tool to directly identify CHC could be a reliable tool in a “test and treat” strategy.

## Introduction

Chronic hepatitis C (CHC) affects about 80 million individuals worldwide [1], and in 2013, its complications, namely cirrhosis and liver cancer, have caused over 700,000 deaths [2]. Africa is one of the most endemic regions with about 19 million seropositive individuals [3].

The new all-oral antiviral treatments have greatly simplified the management of hepatitis C virus (HCV)-infected patients and made HCV elimination conceivable. But the current lack of diagnostic facilities limits access to CHC care. Indeed, an estimated 85% of HCV-infected individuals are unaware of their infection [4], especially in low and middle income countries (LMIC) where 75% of the HCV-individuals live [1].

The standard chronic hepatitis C (CHC) diagnostic algorithm is based on screening by anti-HCV antibody detection (HCV-*Ab*), using enzyme-linked immunosorbent assays (ELISA), supplemented by the detection of HCV RNA by nucleic acid amplification techniques (NAATs) to confirm viral replication. Both methods require highly skilled human resources and molecular technology that are available only in centralized laboratory structures [5]. Furthermore, NAATs are expensive, resulting in a high proportion of patients who tested positive for HCV-*Ab* lost to follow-up. Consequently, as CHC usually remains asymptomatic, most patients are diagnosed at late stages of CHC evolution, with an increased risk of mortality. Simplifying this diagnostic procedure is therefore crucial to scaling up access to CHC care [6,7].

The HCV core antigen (cAg) has been proven to be a good marker of HCV replication. Indeed, unlike HCV-*Ab*, cAg can be detected during the seroconversion phase [8–10] and, as HCV RNA, its detection allows for identifying active infection. Moreover, it correlates well with HCV RNA, in various populations: mono-infected [11,12], HIV-HCV co-infected, liver transplanted [13] and hemodialysed patients [14]. In addition, its quantification is faster and two to five times cheaper than a viral load test [15].

Hence, several studies have addressed its potential role as an alternative to RNA quantification in the current two-step diagnostic procedure. But none was conducted in a resource-constrained area nor with a one-step strategy based only on cAg quantification. Moreover, data on the influence of HIV or HBV infection, both chronic infections prevalent in LMIC, is lacking on its diagnostic performance [16].

The present cross-sectional validation study aims to assess the performance of the ARCHITECT HCV Ag assay (Abbott Diagnostics) in differentiating patients with CHC from HCV-negative individuals, in comparison to the current diagnostic algorithm, and to evaluate the impact of cofactors such as HBV and HIV status on its diagnostic accuracy.

## Materials and methods

### Study samples

The samples, extracted from the serum bank of the Pasteur Center in Cameroon (PCC), were drawn from individuals in various medical settings throughout Cameroon and sent to the PCC for HCV, HIV or HBV testing from January 2013 to April 2015. They all had provided consent for their samples to be stored and used for future research.

The protocol of the present study was approved by the National Ethics Committee for Human Health Research of Cameroon.

Group A included sera from individuals with CHC (positive HCV-*Ab* ELISA serology and quantifiable HCV RNA). Group B comprised sera that was negative for CHC (HCV-*Ab* ELISA serology, either negative or positive with undetectable HCV RNA).

All samples have been tested for the presence of HBsAg and HIV-antibodies. Age and gender were retrieved from the PCC database, as well as the HCV genotype when available. When missing, HCV genotyping was performed. Because the remaining sample volume after the cAg test was often too low, only 132 samples had their genotype determined.

### Laboratory methods

#### HCV core antigen quantification

Quantification was performed using a fully automated chemiluminescent microparticle immunoassay (CMIA) (ARCHITECT HCV antigen assay; Abbott Diagnostics, Chicago, Illinois). A threshold of 0.5 Log fmol/L and an indeterminate zone between 0.50 Log fmol/L and 1 Log fmol/L were used. Indeterminate samples were retested twice and considered nonreactive if both supplementary tests were negative; otherwise they were considered reactive.

#### Anti-HCV Ab detection

The third-generation ELISA ARCHITECT HCV *Ab* assay (Abbott Diagnostics, Wiesbaden, Germany) was used. This test uses recombinant antigens, allowing for the detection of serum antibodies against the core protein and the non-structural proteins NS3, NS4 and NS5. The results are calculated with a formalized signal, according to a threshold value.

#### HCV RNA quantification and genotyping

The Abbott RealTi*m*e HCV assay was used. This assay has a lower limit of detection of 12 IU/ml [17].

HCV genotyping and subtyping were performed by amplification, sequencing and phylogenetic analysis of a 382-nt fragment of the NS5B gene and a 360-nt fragment of the core gene as described elsewhere [18].

#### HIV serology

The fourth-generation CMIA ARCHITECT HIV *Ab*/Ag Combo assay (Abbott Diagnostics, Wiesbaden Germany) and ELISA HIV-1/2Genscreen Ultra Ac/Ag (BioradMarnes-La-Coquette, France) were used as first and second-line tests, respectively. Samples reactive for both tests were classified as positive and referred for HIV serotyping using an in-house ELISA.

#### HBsAg serology

The fourth-generation CMIA ARCHITECT HBsAg qualitative assay (Abbott Diagnostics, Wiesbaden Germany) was used. Reactive samples were re-tested using ARCHITECT HBsAg qualitative confirmation assay.

All the assays were conducted according to the manufacturer’s instructions and, except for cAg quantification, at the PCC. A Cameroonian technician from the PCC performed the cAg quantification at the Mondor Hospital in France.

### Statistical analysis

Using a previously described method [19], the study was powered in order to test desirable levels of the pair [false positive fraction (FPF), true positive fraction (TPF)] at (0.02, 0.95). Non-inferiority criteria were then selected with minimally acceptable values of FPF and TPF at (0.05, 0.85), respectively. Our aim was to test a one-sided, null hypothesis assuming a joint power of 0.90 and type I error of 0.05. As the HCV prevalence in Cameroon presents a cohort effect [20] (30% among individuals aged over 50, 5% for those under it), a weighted prevalence has been used, based on the number of expected individuals in each age strata (60% and 40%, respectively). When accounting for this weighted prevalence of 0.20 and correcting calculations on a 90% probability that the sample obtained will be at least as large as required, the minimum number of participants needed was 555 and 476 (for group A and group B, respectively). Since both FPF and TPF were considered, the joint 95% confidence region was given from the 97.5% univariate intervals. To facilitate clinical interpretation, we have reported the sensitivity (TPF) and specificity (1-FPF). The final sample size is lower than estimated here, but the lower limits of the sensitivity and specificity confidence intervals obtained are higher than the minimally acceptable values, thus we can reject H0 and conclude that the test meets the minimal performance criteria.

The association between cAg levels and categorical variables was evaluated by the Kruskal-Wallis test or Wilcoxon test, while the association with continuous variables was compared by the Spearman test. The level of significance of the correlation between cAg levels and HCV RNA quantification was calculated using Spearman’s correlation. All differences were considered significant for a *p* value ≤0.05.

The cAg quantification test was compared to the diagnostic algorithm used to define group A and B. Sensitivity (Se), specificity (Sp), positive and negative predictive value (PPV and NPV, respectively), and positive and negative likelihood ratio (LR+ and LR–, respectively) were estimated. ROC curves were plotted and their corresponding area under the curve (AUC) was calculated in each patient population (HCV mono-infected, HBV- or HIV-co-infected); AUCs were compared using the method described by DeLong (18). The optimal detection threshold, defined as the cutoff value associated with the highest proportion of correctly classified patients in the overall study population and maximizing both sensitivity and specificity, was determined from the corresponding ROC curve.

Statistical analyses were performed using STATA (v11.2, College Station, TX, USA) statistical software.

## Results

### Study samples

A total of 1037 samples were initially selected. Among them, 28 were excluded from the statistical analysis for the following reasons: 11 were tri-infected with HIV, HBV and HCV and did not represent a large enough group to be analyzed, 7 had an unknown HIV and HBV status and 10 had a first result in the indeterminate zone and no additional serum was available for the retest. Thus, 1009 samples have been included in the analysis; their characteristics are shown in Table 1. Among the 544 samples from group A, 489 (89.9%) were HCV mono-infected, 27 (5.0%) were HIV-HCV co-infected and 28 (5.1%) were HBV-HCV co-infected. Group B was comprised of 465 samples: 335 (72.0%) un-infected, 51 (11.0%) HIV-infected and 79 (17.0%) HBV-infected.

**Table 1.** Demographic and virological characteristics of the study population

	Group A (*n* = 544)			Group B (*n* = 465)		
	HCV-positive	HIV-positive	HBV-positive	No infection	HIV-positive	HBV-positive
	(*n* = 489)	(*n* = 27)	(*n* = 28)	(*n* = 335)	(*n* = 51)	(*n* = 79)
**Female, *n* (%)**	251 (51.3)	12 (44.4)	14 (50.0)	194 (57.9)	30 (58.8)	41 (51.9)
**Age, mean (SD)**	59.8 (0.51)	57.3 (1.7)	54.9 (2.2)	40.8 (0.95)	40.6 (1.7)	35.5 (1.4)
**Virology, median (IQR)**						
HCV RNA (Log IU/mL)	6.0 (0.86)	6.2 (1.4)	5.8 (1.6)	NA	NA	NA
HCV cAg (Log fmol/L)	2.9 (1.3)	3.0 (2.0)	2.7 (1.4)	<0.5	<0.5	<0.5
**Genotype, *n* = 132, *n* (%)**						
1	45 (37.2)	2 (40.0)	2 (33.0)	NA	NA	NA
2	39 (32.2)	1 (20.0)	1 (17.0)	NA	NA	NA
4	37 (30.6)	2 (40.0)	3 (50.0)	NA	NA	NA

NA: not applicable.

The mean patient age was 50.4 ± 16.9 years with a significant difference between group A (mean age: 59.4 ± 11.2) and group B (mean age: 39.9 ± 16.4) (*p* < 0.00001), consistent with the cohort effect observed in the HCV prevalence in Cameroon (17).

In group A, the median cAg level was 2.9 Log fmol/L (IQR = 1.3), 3.0 Log fmol/L (IQR = 2.0) and 2.7 Log fmol/L (IQR = 1.4) in HCV mono-infected, HIV- and HBV-co-infected patients, respectively. Likewise, the median HCV RNA level was 6.0 Log IU/mL (IQR = 0.86), 6.2 Log IU/mL (IQR = 1.4) and 5.8 Log IU/mL (IQR = 1.6).

### Correlation between cAg and HCV RNA levels (group A)

**Figure 1.**
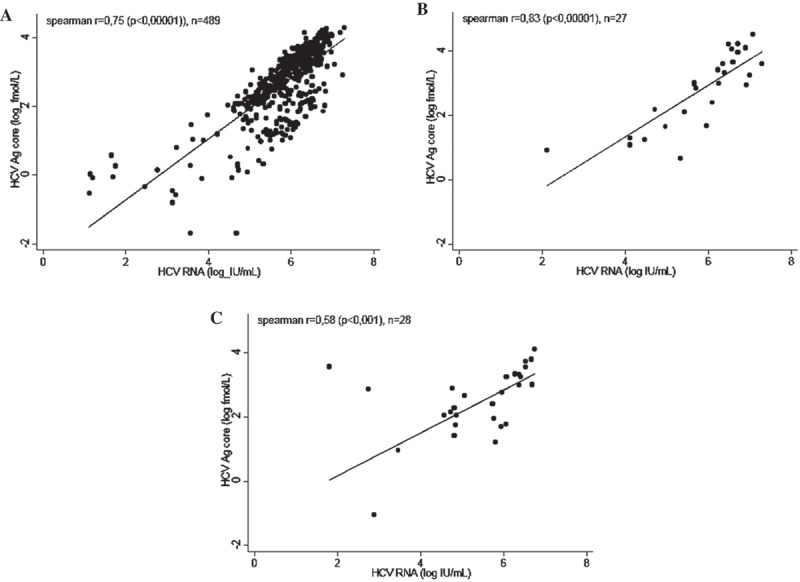
Correlation between cAg and HCV RNA in mono-infected (a), HIV-infected (b) and HBV-infected (c) sera.

**Figure 2.**
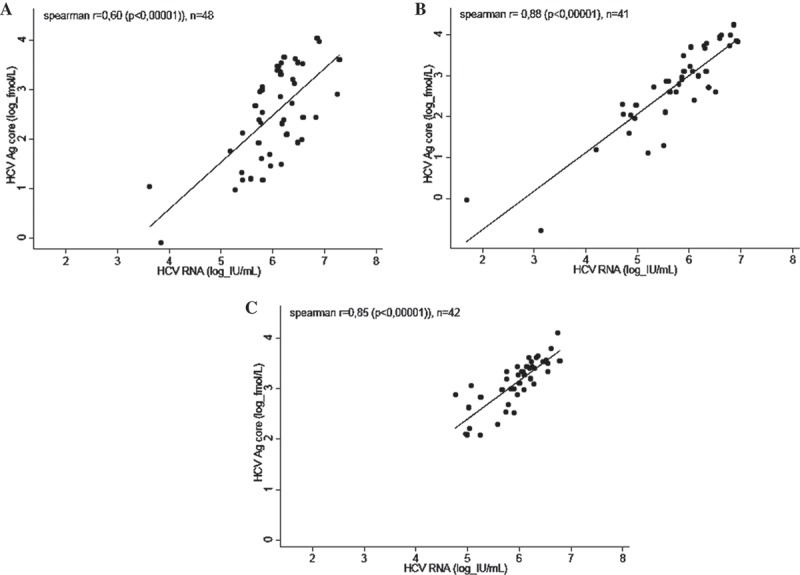
Correlation between cAg and HCV RNA in genotype 1 (a), genotype 2 (b) and genotype 4 (c) sera.

A significantly positive correlation between cAg and HCV RNA levels was observed for HCV mono-infected patients (*r* = 0.75, *p* < 0.00001, *n* = 489), HIV-HCV co-infected patients (*r* = 0.84, *p* < 0.00001, *n* = 27) and HCV-HBV co-infected patients (*r* = 0.58, *p* < 0.001, *n* = 28) (Figure 1). This correlation was significant for all genotypes (Figure 2) with correlation coefficients of *r* = 0.60 (*p* < 0.00001), *r* = 0.88 (*p* < 0.00001) and *r* = 0.85 (*p* < 0.00001) for genotype 1 (*n* = 48), 2 (*n* = 41) and 4 (*n* = 42), respectively. Coefficient values did not differ when the outliers visible on the graphics were withdrawn.

### Variables associated with cAg levels (group A)

No significant association was found between cAg quantification and age (*p* = 0.16), gender (*p* = 0.88), and infection with HBV (*p* = 0.4) or HIV (*p* = 0.3). In genotype 1, the cAg level was significantly lower than in genotype 4 sera (*p* = 0.002). No difference was noted for cAg levels between genotypes 1 and 2 (*p* = 0.10) or 2 and 4 (*p* = 0.2). Insufficient data on HCV genotype in HCV-HIV and HCV-HBV infected patients were available to study this relationship according to the infectious status.

### Diagnostic performance of cAg

In HCV mono-infected sera, the test showed a sensitivity of 95.7% [CI 97.5%: 93.2; 97.5] and a specificity of 99.7% [CI 97.5%: 98.1; 100] corresponding to an AUC of 0.99 [CI 95%: 0.98–1.0].

**Figure 3.**
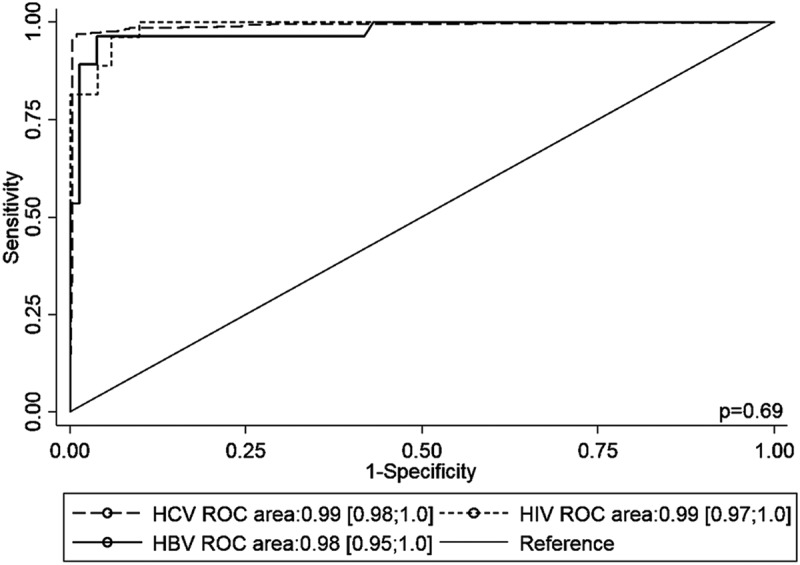
ROC curves of the performance of cAg quantification for the diagnosis of chronic hepatitis C in HCV mono-infected, HIV-infected and HBV-infected patients.

Among HIV- and HBV-infected patients, a sensitivity of 100% [CI 97.5%: 85.0; 100] and 96.4% [CI 97.5%: 79.2; 99.9]; a specificity of 88.2% [CI 97.5%: 74.3; 96.2] and 96.2% [CI 97.5%: 88.1; 99.4]; and an AUC of 0.99 [CI 95%: 0.97; 1.0] and 0.98 [0.95; 1.0], were observed, respectively (Table 2). No significant difference was noted between the three AUCs (chi squared test, *p* = 0.69) (Figure 3).

**Table 2.** Performance of cAg quantification by infection group

	*n*	Se [CI97.5%]	Sp [CI97.5%]	PPV^a^	NPV^a^	AUC [CI95%]	LR+	LR–
**Mono**	824	95.7 [93.2; 97.5]	99.7 [98.1; 100]	98.1	99.3	0.99 [0.98–1.0]	319	0.043
**HIV**	78	100 [85.0; 100]	88.2 [74.3; 96.2]	57.6	100	0.99 [0.97–1.0]	847	0
**HBV**	107	96.4 [79.2; 99.9]	96.2 [88.1; 99.4]	80.2	99.4	0.98 [0.95–1.0]	25	0.037

^a^Estimated HCV prevalence in Cameroon: 13.8%.

The test performance did not differ according to genotype, with a sensitivity of 97.9% [CI 97.5%: 87.4–1.0] in genotype 1, 95.1% [CI 97.5%: 81.6–99.6] in genotype 2 and 100% [CI 97.5%: 90.1–100] in genotype 4 (Table 3).

**Table 3.** Performance of cAg quantification by genotype

	*n*	Se [CI 97.5%]	Sp [CI 97.5%]	PPV^a^	NPV^a^	AUC [CI 95%]	LR+	LR–
**Genotype 1**	513	97.9 [87.4–1.0]	97.8 [95.8–99.1]	98.1	99.6	0.99 [0.98–1.0]	326	0.021
**Genotype 2**	506	95.1 [81.6–99.6]	97.8 [95.8–99.1]	87.4	99.2	0.99 [0.96–1.0]	43	0.050
**Genotype 4**	507	100 [90.1–100]	97.8 [95.8–99.1]	87.9	100	0.99 [0.99–1.0]	45	0

^a^Estimated HCV prevalence in Cameroon: 13.8%.

### Optimal detection threshold

The optimal detection threshold was set at 0.53 Log fmol/L, which provided a sensitivity of 95.7%, a specificity of 99.7% and a percentage of correctly classified sera of 97.3% in HCV mono-infected individuals. In HIV and HBV-co-infected patients, this threshold was associated with a sensitivity of 100% and 96.4%, a specificity of 88.2% and 96.2% and a percentage of correctly classified sera of 92.3% and 96.3%, respectively.

### Discordant results

Overall, 32 samples (3.2%) were incorrectly identified by the cAg test, of which 22 were false negative (FN) and 10 false positive (FP). The characteristics of these individuals are reported in Table 4.

**Table 4.** Characteristics of the sera with a discordant cAg quantification result

Diagnosis	Gender	Age	HCV RNA (Log IU/mL)	Ag core (Log fmol/L)	Genotype
**False negative**					
Mono	F	66	2.8	0.16	–
Mono	M	51	1.1	0.033	–
Mono	M	53	5.2	0.42	–
Mono	M	66	4.7	0.33	–
Mono	M	72	3.1	−0.44	–
Mono	M	65	3.8	−0.09	–
Mono	M	59	1.2	−0.07	–
Mono	F	59	1.1	−0.54	–
Mono	M	73	3.1	−0.80	2
Mono	F	57	4.9	0.09	–
HCV-HBV	F	60	2.9	−1.04	–
Mono	M	65	1.7	0.27	–
Mono	M	70	1.7	−0.46	2
Mono	F	63	3.2	−0.57	–
Mono	M	53	4.7	0.15	–
Mono	M	56	2.5	−0.35^a^	–
Mono	M	40	3.6	0.29	–
Mono	M	53	3.6	−1.7	–
Mono	M	57	4.7	−1.7	–
Mono	M	48	4.6	−0.066	–
Mono	M	69	4.7	0.27	–
Mono	M	56	5.3	0.33	–
**False positive**					
HIV	F	46	–	0.85^a^	
HIV	M	46	–	0.53^a^	
HIV	F	55	–	1.6	
HIV	M	46	–	1.4	
Un-infected	F	61	<1.0^b^	3.7	
HBV	F	46	<1.0^b^	2.6	
HBV	M	44	<1.0^b^	1.4	
HIV	M	32	<1.0*	0.77^a^	
HBV	M	41	<1.0*	1.2	
HIV	M	58	<1.0*	1.2	

^a^Retested.

^b^Undetectable viral load.

The FN individuals had HCV-RNA levels significantly lower than true positive (TP) individuals (Wilcoxon test, *p* = 0.00001), with a median of 3.4 Log UI/mL (IQR = 2.3) in FN sera and 6.1 Log UI/mL (IQR = 0.8) in TP sera. Likewise, the gender repartition in the two groups was significantly different: 77.3% of FN sera came from men compared to 47.9% in TP sera (Fisher test, *p* = 0.008).

The FP and true negative sera differed significantly only in their proportion of HIV-infected sera, which was higher in FP sera: 60% versus 9.9% (Fisher test, *p* < 0.0001).

## Discussion

The lack of affordable and adapted viral diagnostic tests in resource-limited countries represents a barrier to implementing CHC diagnosis and, hence, to ensuring HCV treatment access. The ANRS 12,336 project is the first validation study with sufficient power to demonstrate the excellent diagnostic performance (se = 95.7%, sp = 99.7%) of the cAg quantification assay (Abbott ARCHITECT) in detecting CHC in patients from Sub-Saharan Africa. Smaller studies conducted in Europe and the USA have reported similar results with a sensitivity from 90.9% to 98.1% and a specificity from 98.2% to 100% [12,21,22], depending on the study. Contrary to these previous studies, this study aimed to compare the cAg quantification used as a one-step procedure to the current two-step one and not only to RNA quantification. A one-step strategy of this type could make the diagnostic process simpler, faster and less expensive, thus increasing access to CHC diagnosis and the linkage to care [23].

This study showed that cAg and HCV RNA are significantly correlated in both mono-infected and co-infected patients, as suggested by previous studies [24–27], and that cAg can therefore be used as a surrogate to HCV RNA in these populations. Moreover, high classification probabilities have been observed in sera infected with HBV or HIV. Previous studies in HIV-positive sera have already reported high sensitivity and high specificity (100% and 95% to 97.9%, respectively) [27–29]. One study reported a specificity closer to the one found in our study (87.5%) [30]. The excellent diagnostic performance of cAg quantification in HBV-infected patients is clearly an advantage in low-resource countries where the prevalence of HBsAg carriage is high. To our knowledge, only one study has evaluated the influence of HBV infection on the assay performance (*n* = 57 patients) and reported a non-significant correlation between HCV RNA and cAg levels (*r* = 0.04, *p* = 0.822) but a sensitivity of 100% and a specificity of 90.9% [31]. These results are consistent with ours: low correlation and good discriminatory probabilities.

Moreover, the validity of the detection threshold given by the manufacturer was confirmed: the estimated optimal detection cutoff did not improve the percentage of sera correctly classified in HCV mono-infected patients and increased it by not more than 1% in HIV- and HBV-infected patients.

Finally, no influence of age, gender and genotype was found on the test performance, which is consistent with other published studies [28,30,31].

The sensitivity of the test was lower in patients with a low HCV viral load; this was expected because of the lower limit of detection of the test corresponding to about 3 Log UI/mL HCV RNA [24,28]. However, since 95% of the patients infected by HCV usually have HCV-RNA higher than this limit [32–34], it should not affect the potential of the test as a diagnostic tool.

Several limitations in this study should be acknowledged. First, it lacks power to assess the influence of concomitant HIV or HBV infections on the test performance; studies with a higher number of co-infected patients should therefore be conducted. It is however nearly impossible in view of logistical constraints. Furthermore, all other published studies concluded that HIV and HBV co-infections have no impact on the diagnostic performance of cAg quantification. Second, we did not perform it in real-life settings but retrospectively in a laboratory.

Despite its excellent performance, the implementation of cAg quantification on a large scale in resource-limited countries presents some limitations in the present time. First, it requires a fully equipped laboratory and a trained staff; yet these conditions are often limited to big cities in resource-limited countries. This implies that patients need to travel to be diagnosed or that a high quality sample transportation system must be implemented. The dried blood spot (DBS) technology – a filter paper where blood samples are dried and can be easily sent off to laboratories – could make cAg testing available even in remote regions [35]. However, a recent study showed that the cAg quantification test sensitivity was considerably reduced after using DBS, whereas the performance of HCV RNA detection on DBS was satisfactory [36]. Thus, for now, DBS can be implemented only combined with molecular tests, which are still very expensive in the majority of resource-limited countries, resolving the issue of decentralization but not that of the diagnostic cost. An alternative to DBS for decentralized CHC diagnosis are the point-of-care (POC) tests, which can perform diagnostic testing directly at the bedside. Several POC tests for HCV are currently in development, using either cAg quantification or molecular testing, and should be available for marketing towards the end of 2017 [23]. Lastly, the cost of the cAg quantification makes it an economical replacement for viral load tests but, since it is still three times more expensive than HCV-*Ab* detection (PCC personal communication), its price may be a limitation to its use as a one-step diagnostic procedure. This may be overcome in countries or among high risk groups where CHC prevalence is high enough to make the use of cAg quantification more cost-effective than a two-step procedure combining serology and HCV PCR in patients with positive HCV antibodies.

## Conclusions

In view of its diagnostic performance, cAg quantification could replace the current two-step CHC diagnostic approach. But, until some POC tests are developed or the quality of its detection on DBS is improved, cAg will remain unsuitable for an HCV diagnostic strategy on a large and decentralized scale. Nevertheless, it can serve as a confirmatory test in the current diagnostic procedure at a lower price than NAATs, and thus contribute to scaling up access to HCV diagnosis. Further cost-effectiveness studies are necessary to determine in which settings these different strategies (one-step versus two-step, with RNA or cAg quantification, laboratory or POC tests) would be cost-effective.

Furthermore, its good performance as a detection test suggests that cAg quantification may also be used as a monitoring tool for treatment efficacy instead of PCR, as recently suggested [37]. The second part of this project contained within the ANRS 12,311 TAC (NCT02405013) trial [38] that is currently assessing the feasibility of interferon-free therapy in Sub-Saharan Africa will provide further insights about this possible role.

## Abbreviations

AUC, area under the curve; cAg, HCV core antigen; cDNA, complementary DNA; CHC, chronic hepatitis C; CMIA, chemiluminescent microparticle immunoassay; DBS, dried blood spot; DNA, deoxyribonucleic acid; EIA, enzyme immunoassay; FPF, false positive fraction; HBV, hepatitis B virus; HCC, hepatocellular carcinoma; HCV, hepatitis C virus; HCV-*Ab*, anti-HCV antibody; HIV, human immunodeficiency virus; IQR, interquartile range; LMIC, low and middle income countries; LR+, positive likelihood ratio; LR–, negative likelihood ratio; NAATs, nucleic acid amplification techniques; NPV, negative predictive value; PCC, Pasteur Center in Cameroon; PCR, polymerase chain reaction; POC, point of care; PPV, positive predictive value; RNA, ribonucleic acid; ROC, receiver operating characteristic; Se, sensitivity; Sp, specificity; TPF, true positive fraction.
